# Antenatal Hydronephrosis: Differential Diagnosis, Evaluation, and Treatment Options

**DOI:** 10.1100/tsw.2006.366

**Published:** 2006-03-05

**Authors:** C.D. Anthony Herndon

**Affiliations:** University of Alabama at Birmingham, AL, USA

**Keywords:** antenatal hydronephrosis, pediatrics

## Abstract

The diagnosis, evaluation and management of antenatal hydronephrosis has undergone a two stage paradigm shift since the advent of prenatal ultrasonography in the early 1980s. Initially the identification of a large number of asymptomatic infants appeared to afford the surgeon the opportunity for preemptive intervention. However, it has now become apparent that antenatal hydronephrosis (AH) is far more difficult to interpret thanoriginally perceived. The initial enthusiasm for surgery has now been replaced by a much more conservative approach to ureteropelvic junction(UPJ) obstruction, multi-cystic dysplastic kidney(MCDK), vesicoureteral reflux and the non-refluxing megaureter. This review will highlight the postnatal evaluation of AH and include an overview of the Society for Fetal Urology grading system for hydronephrosis. The differential diagnosis and treatment options for UPJ obstruction, vesicoureteral reflux, MCDK, duplication anomalies, megaureter, and posterior urethral valves will be discussed.

### INTRODUCTION

The detection of urinary anomalies has changed significantly since the inception of fetal sonography. The mechanism of detection of urinary anomalies has shifted over the last 25 years from the symptomatic patient that may present with a palpable mass, urinary tract infection, or hematuria to the asymptomatic patient that presents with antenatal hydronephrosis (AH). Routine screening ultrasounds are typically performed in the U.S. during the 18$^{th}$- to 20$^{th}$-week gestational age. Although fetal renal development begins early in the 5$^{th}$ gestational week, the ability for fetal sonography to detect renal anomalies does not begin until the early 2$^{nd}$ trimester. Appropriate fetal follow-up is based on signs of upper and lower urinary obstruction, in addition to other organ system involvement. Although rare, fetal intervention does play a role for some patients, however, no reliable predictors of renal outcome have stood the test of time. Although the differential is broad, ureteropelvic obstruction (UPJ) is by far the most common anomaly detected.

The postnatal evaluation is performed in the neonatal period and should include a thorough evaluation of the upper and lower urinary tract. In the absence of suspected bladder outlet obstruction, the infant may leave the hospital shortly after birth. Obstructive (UPJ, megaureter, posterior urethral valves [PUV], duplex system, cloaca, megalourethra, prune belly syndrome [PBS]) and nonobstructive (megaureter, mild UPJ, vesicoureteral [VUR]) lesions will be identified with the implementation of a thoughtful postnatal evaluation (Figs. 1 and 2). It is clear that the perceived need for intervention for most of the common disorders detected antenatally, such as UPJ and megaureter obstruction, has decreased over the last 2 decades. The purpose of this review is to better clarify these issues and develop a contemporary algorithm to manage AH.

Ureteropelvic junction obstruction
Multicystic dysplastic kidney
Megaureter
Vesicoureteral reflux
Ureterocele
Ectopic ureter
Ureteral stricture
Ureteral diverticuli
Posterior urethral valves
Prune belly syndrome
Urethral atresia
Megacystis-megaureter microcolon
VACTERL syndrome
Cloacal anomaly

**FIGURE 1**. Differential diagnosis of AH.

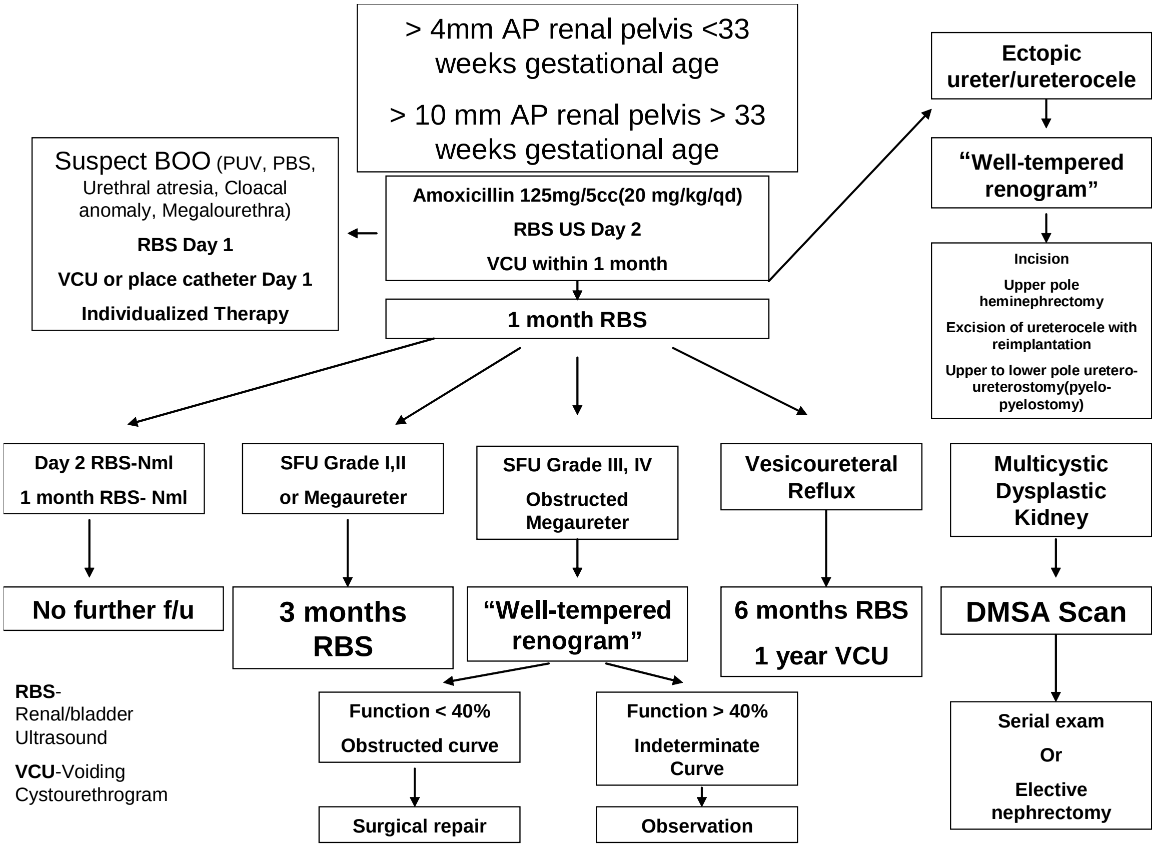

**FIGURE 2.** Postnatal evaluation of AH.

## FETAL DETECTION AND FOLLOW-UP

### Fetal Ultrasound

The accepted standard for anterior posterior (AP) pelvic dilation considered to be significant is based on the original work by Corteville et al.[6]. In their series, a renal pelvis AP diameter of >4 mm at 33 weeks gestational age (GA) or >7 mm at 40 weeks GA demonstrated a sensitivity of 100% for the identification of those patients with abnormal renal function or those that required subsequent intervention postnatally. With these data, Corteville identified an upper-limit threshold of AH that warrants postnatal evaluation.

Other centers have offered an alternate view by identifying a lower-limit threshold for patients with nonsignificant prenatal renal pelvis dilation. Siemens et al. demonstrated prenatal renal pelvis AP thresholds of <6 mm (<20 weeks GA), <8 mm (20–30 weeks GA), and <10 mm (>30 weeks GA) to be predictive of those patients that did not manifest postnatal urologic disease[46].

In addition to characterizing hydronephrosis, the antenatal ultrasound should document: the level of amniotic fluid, cycling of the urinary bladder, an attempt at visualization of a ureter, the presence of a contralateral kidney, characterization of renal cysts, and the presence of other organ system abnormalities. When a severe abnormality is detected, the general recommendation is to follow this with 2- to 4-week-interval ultrasound. If a deterioration of the clinical setting occurs, e.g., worsening oligohydramnios, then antenatal intervention with fetal shunting may be indicated.

Fluctuations in fetal renal and ureteral biometrics may occur during serial gestational imaging. Bobrowski et al. reported that minimal pelvic dilation progressed to hydronephrosis defined as >10 mm in 11% of fetuses. Bilateral dilation was more predictive of progression in their population representing 80% of entire cohort that progressed[4]. Additionally, severe AH (>20 mm) may be underrepresented on initial postnatal imaging and subsequently return to prenatal levels necessitating delayed surgical intervention[9]. Other centers have reported that renal pelvis fluctuations associated with fetal voiding, the presence of ureteral dilation, and progression to hydronephrosis may be predictors of VUR reflux[24,43].

When using the accepted standards set forth by the Corteville group (≥4 mm 2$^{nd}$ trimester or ≥7 mm 3$^{rd}$ trimester), AH has been seen in up to 4.5% of all pregnancies. The report from the Brussels Free University Perinatal Nephrology study group revealed that 62% of infants with mild to moderate (<15 mm AP diameter) AH demonstrated a renal anomaly, but only 39% were significant uropathies. Furthermore, with the identification limited to only the 2$^{nd}$ trimester, the detection rate of significant renal disease fell to 12%[28].

The Society for Fetal Urology (SFU) developed a standardized evaluation system designed, in part, to help predict those patients that may need surgical intervention postnatally[14] (Fig. 3). This classification system incorporates collecting system dilation in addition to parenchymal integrity. The system is a spectrum with grade 1 demonstrating normal parenchymal thickness and only renal pelvis splitting, and grade 4 revealing distention of the renal pelvis and calyces in addition to parenchymal thinning (Fig. 4A–D). Limitations exist with the SFU classification scheme; variables such as renal length in relation to GA may improve the predictive value of this system.

### Fetal Intervention for Bladder Outlet Obstruction

The ability to detect bladder outlet obstruction antenatally has afforded the opportunity to intervene prenatally in an attempt to salvage renal and pulmonary function. The classic prenatal finding of bladder outlet obstruction includes: bilateral hydroureteronephrosis, a thickened bladder wall, dilated prostatic urethra, and oligohydramnios (Fig. 5). The most common cause of bladder obstruction in the neonate is posterior urethral valves, but other obstructive disorders include urethral atresia, prolapsing ureterocele, and select cases of prune belly syndrome. A ureterocele represents the most common form of bladder outlet obstruction in the female.

| | |
|---|---|
| Grade 1 | Renal pelvis splitting |
| Grade 2 | Moderate renal pelvis splitting confined to renal border |
| Grade 3 | Significant renal pelvis distention outside of renal border, uniform calyceal distention, renal parenchyma normal |
| Grade 4 | Significant renal pelvis distention, significant calyceal distention, renal parenchyma demonstrates thinning |

**FIGURE 3**. SFU grading system for hydronephrosis.

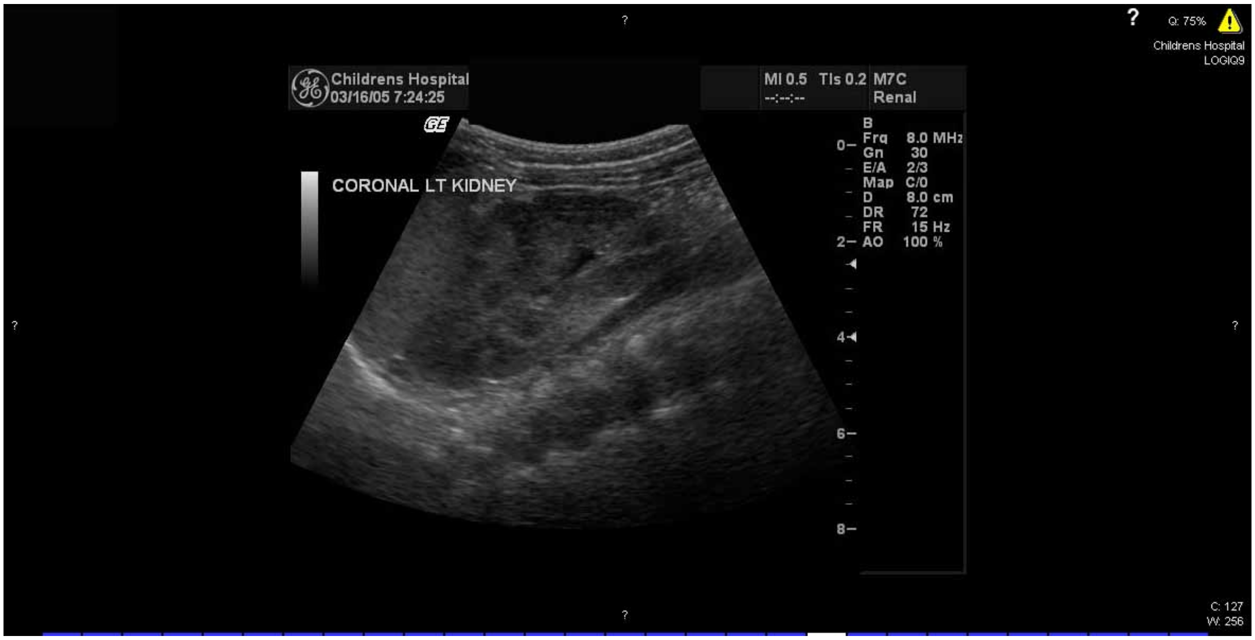

**FIGURE 4A**. Ultrasound demonstrating renal pelvis splitting (SFU grade 1).

The need for antenatal intervention for bladder outlet obstruction has been estimated to be 1:40,000 deliveries. By far, the most common intervention employed is early delivery after confirmation of adequate fetal lung development. To date, no randomized trial exists to determine the true efficacy of intervention for bladder outlet obstruction. It is clear, however, that the only patients who should be considered for intervention are those with oligohydramnios in the presence of bladder outlet obstruction or possibly bilateral upper tract obstruction[23]. Attempts to develop parameters that are predictive of preserving renal function have been successful[29]. However, indicators of poor renal outcome appear to be more predictive. Elevated levels of urinary sodium (>100 mEq/l), chloride (>90 mEq/l), osmolarity (>210 mOsm/l) and B-2 microglobulin may be predictive of unrecoverable renal failure[16,17,29,32]. It is important to note that when assessing renal function via bladder aspiration, several consecutive weekly samples should be obtained because the initial bladder sample is not representative of actual renal function. Elder’s review of fetal shunt intervention revealed that the complication rate was 44% with the most common being shunt migration. More importantly, fetal demise was 79% for the group with oligohydramnios that underwent intervention[11].

Recent long-term follow-up of patients who underwent antenatal intervention for presumptive fetal bladder outlet obstruction have been reported. Johnson and associates from Children’s Hospital of Philadelphia reported the outcomes of 23 antenatal interventions for lower urinary tract obstruction. First-year survival was 91% (21 out of 23) with one death, initially demonstrating a good prognosis on antenatal bladder aspiration. Mean follow-up was 5.8 (1–14) years. At last report, a majority (61%) of patients demonstrated

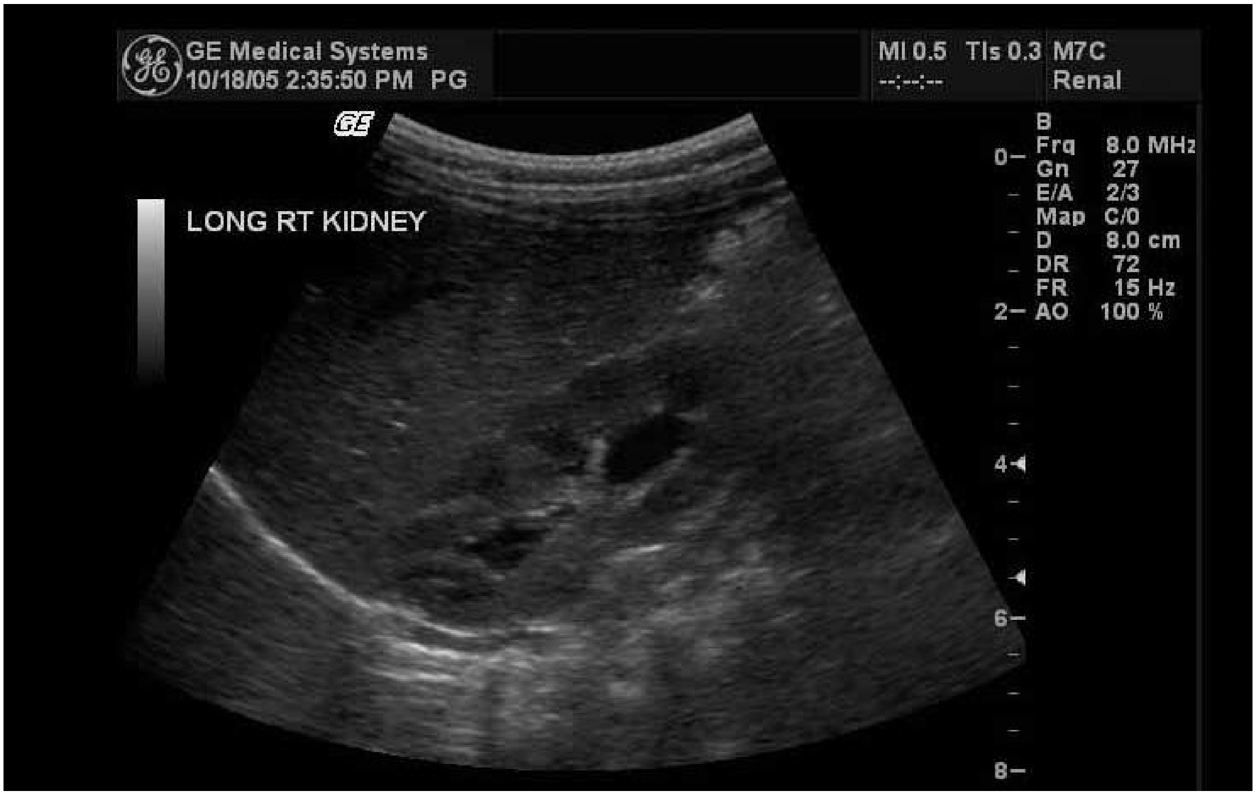

**FIGURE 4B**. Ultrasound demonstrating moderate renal pelvis splitting within renal border (SFU grade 2).

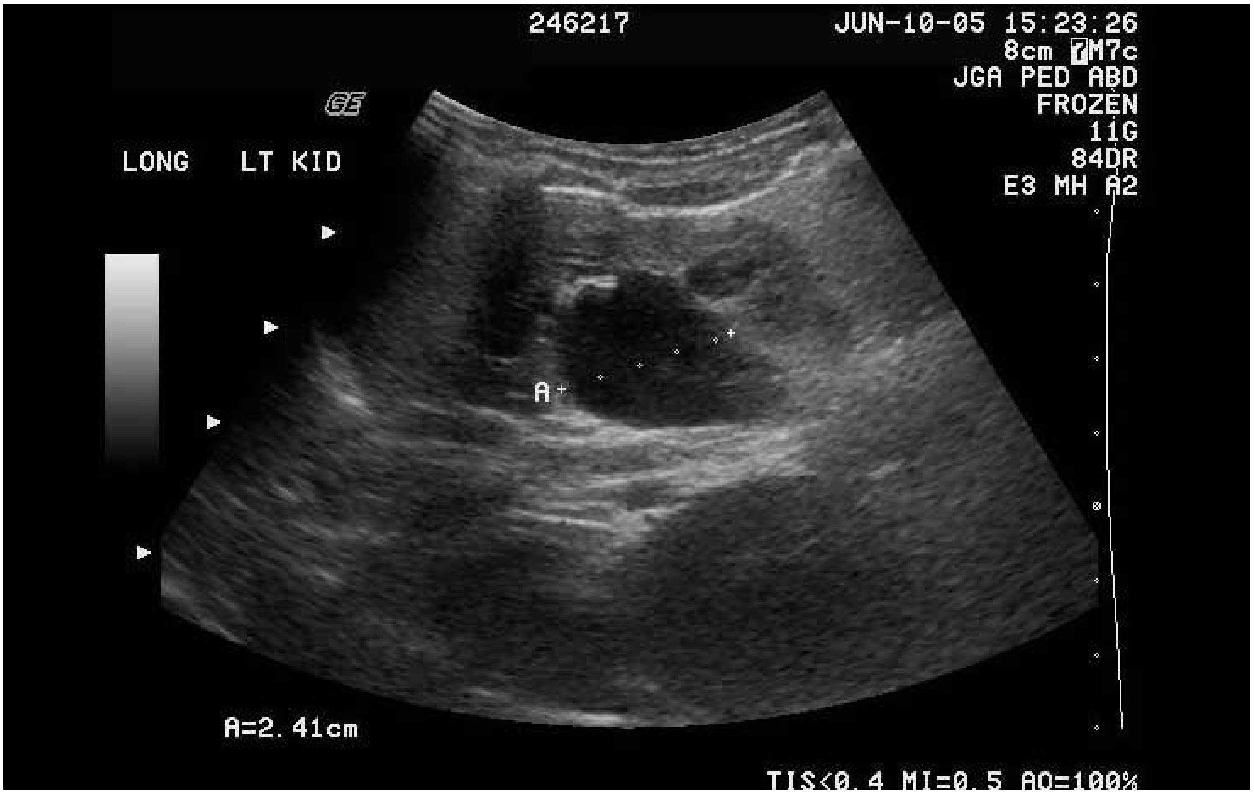

**FIGURE 4C.** Ultrasound demonstrating renal pelvis distention outside of renal border, calyceal distention without parenchymal thinning (SFU grade 3).

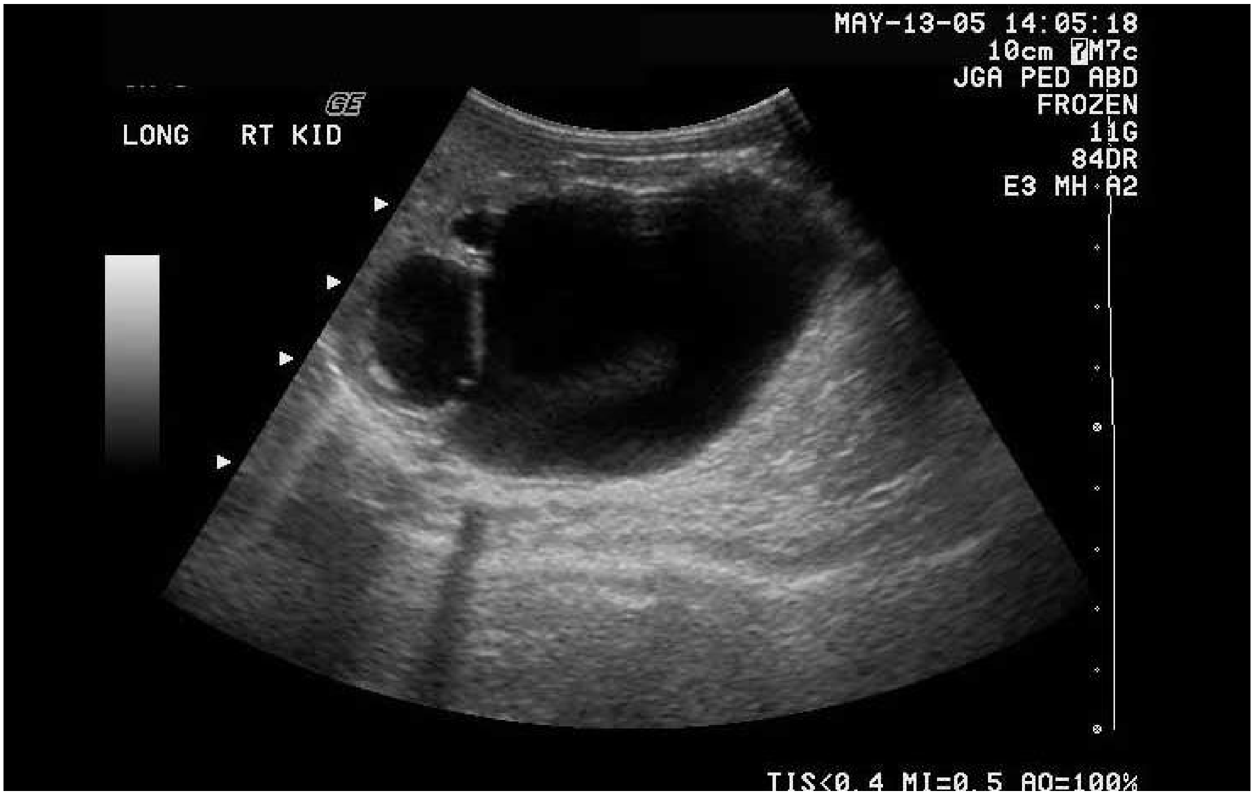

**FIGURE 4D**. Ultrasound demonstrating renal pelvis and calyceal distention with parenchymal thinning (SFU grade 4).

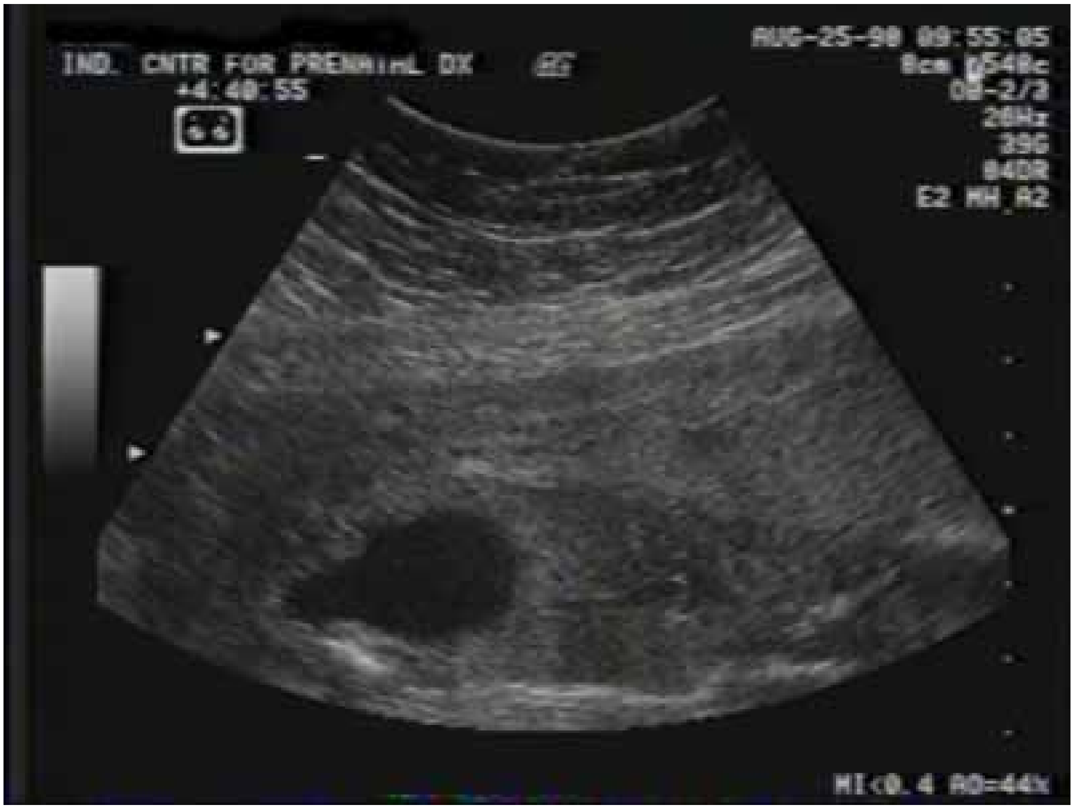

**FIGURE 5.** Antenatal ultrasound of fetus demonstrating “keyhole” sign indicative of the dilated bladder neck, prostatic urethra, and oligohydramnios.

the ability to manage their bladders with spontaneous voiding. Renal function was compromised in most patients with 22% demonstrating mild renal insufficiency and 33% severe renal insufficiency (dialysis). A slight minority (45%) of patients manifest some form of long-term pulmonary disease in the form of asthma or recurrent infections. An interesting finding in this study was the results from the health-related quality of life scores. For the study cohort, the quality of life scores were very similar to standard means available for a healthy population cohort. Furthermore, they scored significantly higher when compared to a chronically ill cohort[3].

Based on the lack of our ability to identify those patients that will benefit from fetal intervention accurately, each clinical scenario should be managed individually. Ideally, a multispecialty team should be utilized that includes a maternal-fetal obstetrician, pediatric urologist, pediatric nephrologists, and neonatologist.

#### Maternal/Fetal MRI

Maternal/fetal magnetic resonance imaging is still in its infancy. Widespread application is limited by cost and access. To date, only a handful of case reports are available in the literature[48]. However, MRI remains an attractive imaging modality because of the absence of fetal radiation exposure and superior resolution when compared to maternal ultrasound.

### POSTNATAL EVALUATION

The postnatal evaluation of AH should include a thorough evaluation of the upper and lower urinary tract. Until the etiology of AH is determined, Amoxil prophylaxis is recommended. A number of imaging modalities including renal ultrasound, voiding cystourethrogram (VCU), and radionuclide renal scan imaging may be required.

#### Evaluation of Bilateral Hydronephrosis in a Male

Any male fetus with antenatal bilateral hydronephrosis and a dilated bladder should be presumed to have posterior urethral valves. Typically SFU grade 3 or 4 hydronephrosis, hydroureter, renal dysplasia, a thickened bladder wall, and low amniotic fluid will exist in the patient with urethral valves, but this is not absolute. The clinician should have a high index of suspicion when evaluating any male with a history of bilateral hydronephrosis. The pediatric urologist should be involved as soon as possible, ideally in the antenatal period.

After delivery, a 5 French feeding tube should be placed in the urinary bladder. A renal ultrasound should be performed on the 1$^{st}$ day of life and the child placed on prophylactic antibiotics. The VCU should be performed to confirm the diagnosis and child transferred to a tertiary center with the feeding tube decompressing the urinary bladder (Figs. 6A–D).

#### Renal/Bladder Ultrasound

Infants are presumed to be relatively dehydrated at birth, which impacts the recommended timing of the initial renal/bladder ultrasound. Dejter and Gibbons reported the development of hydronephrosis in 83% of patients with a initial normal renal ultrasound[8]. In the absence of lower urinary tract obstruction, the initial ultrasound should be performed on the 2$^{nd}$ day of life. Factors such as renal length, AP renal pelvis diameter, presence of renal cyst, renal parenchymal thickness, and ureteral dilation should be measured. It is important to image the urinary bladder as well as upper urinary system during the assessment. For example, a ureterocele resulting in hydronephrosis may be identified in the bladder.

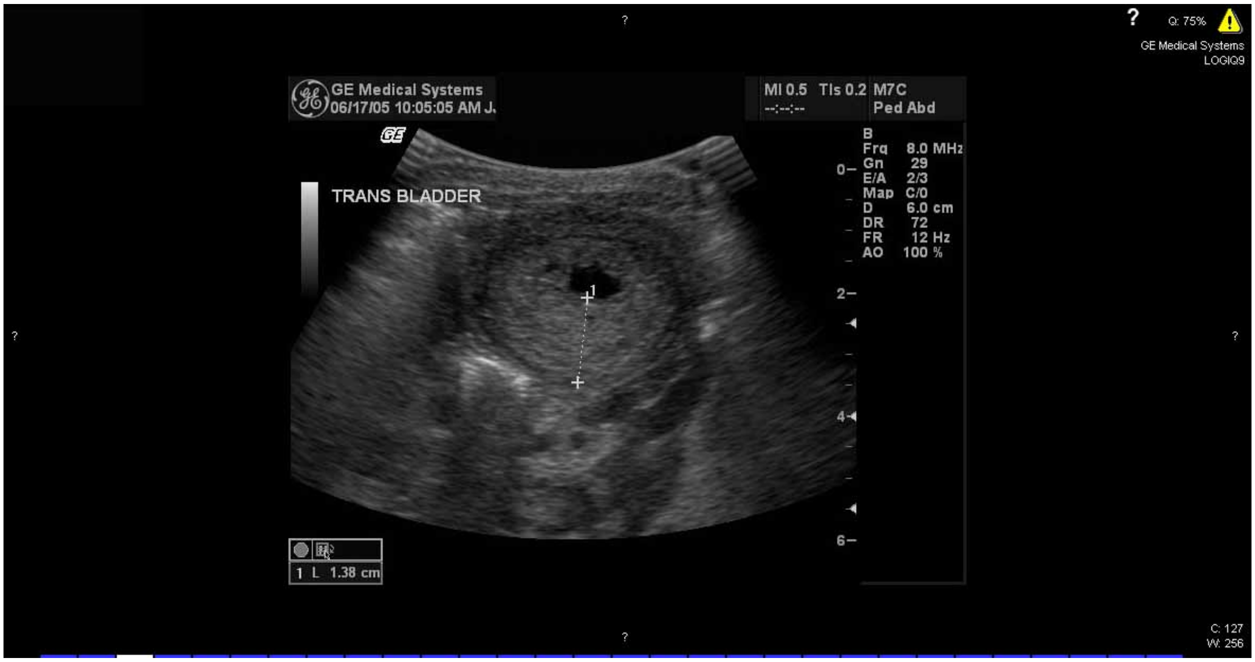

**FIGURE 6A**. Postnatal bladder ultrasound demonstrating a thickened bladder secondary to bladder outlet obstruction from posterior urethral valves.

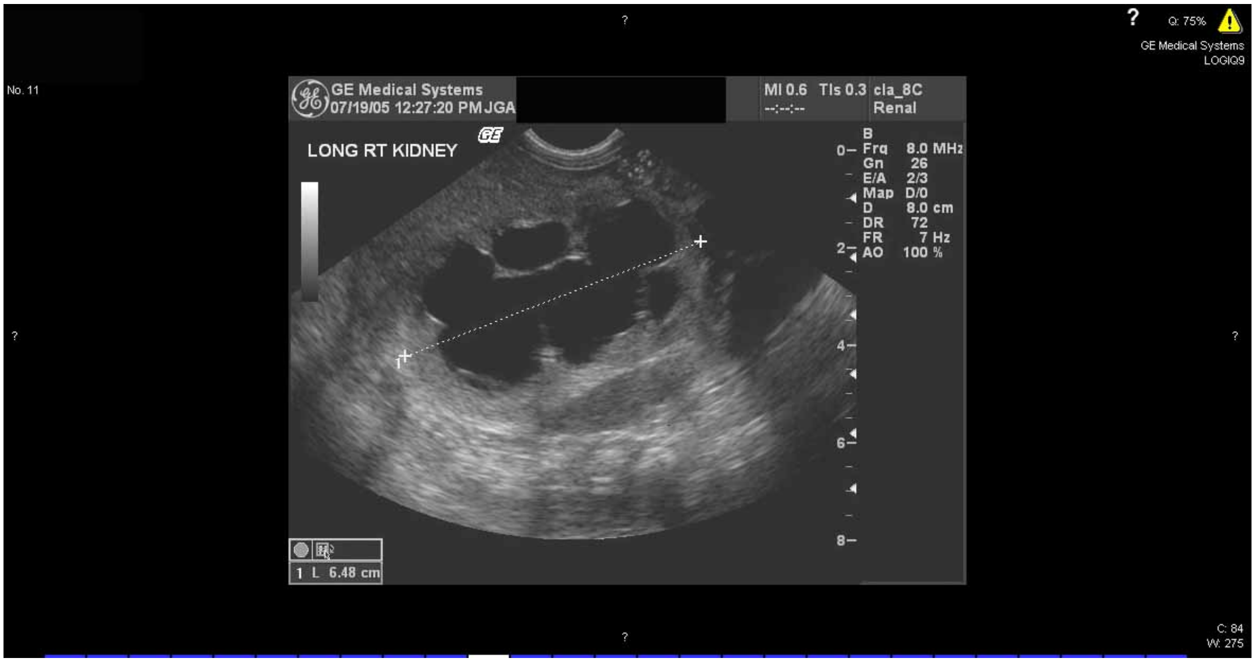

**FIGURE 6B**. Renal ultrasound demonstrating grade 4 hydroureteronephrosis secondary in a patient with posterior urethral valves.

Ultrasound imaging provides adequate anatomic detail in the absence of radiation exposure. However, independently, renal ultrasound is a poor predictor of those patients that will need surgical intervention[21]. In order to standardize evaluations, it is recommended that all images be interpreted with the SFU classification system.

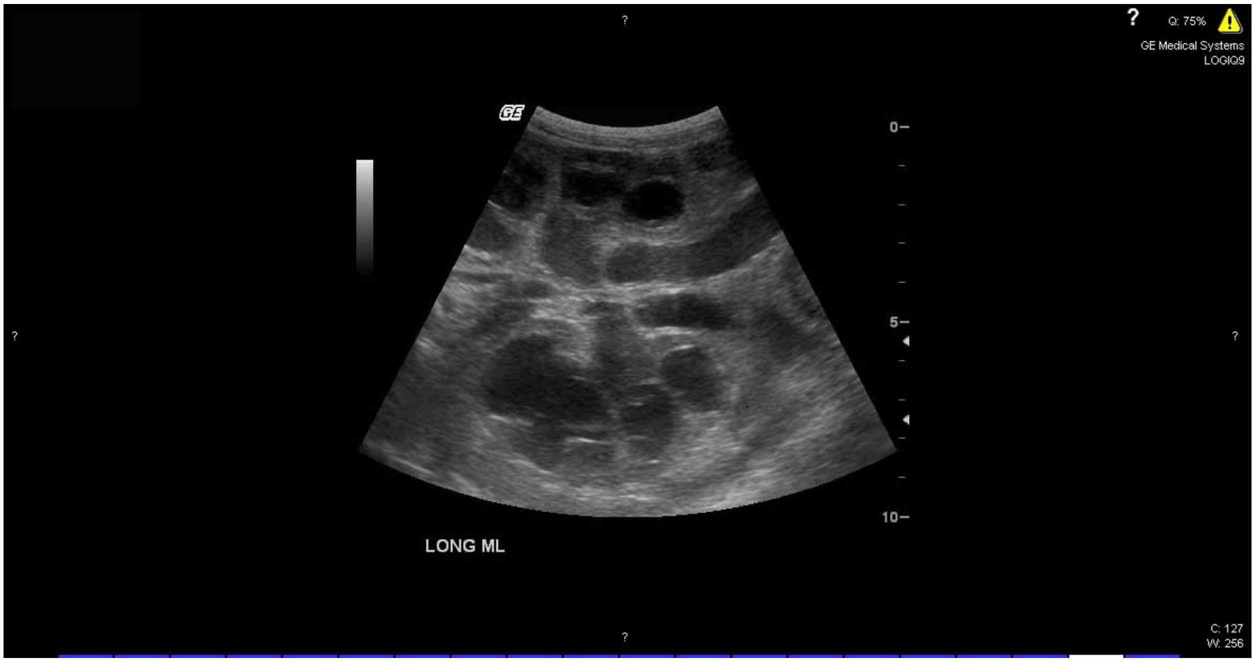

**FIGURE 6C.** Abdominal ultrasound demonstrating bilateral hydroureteronephrosis in a neonate with posterior urethral valves.

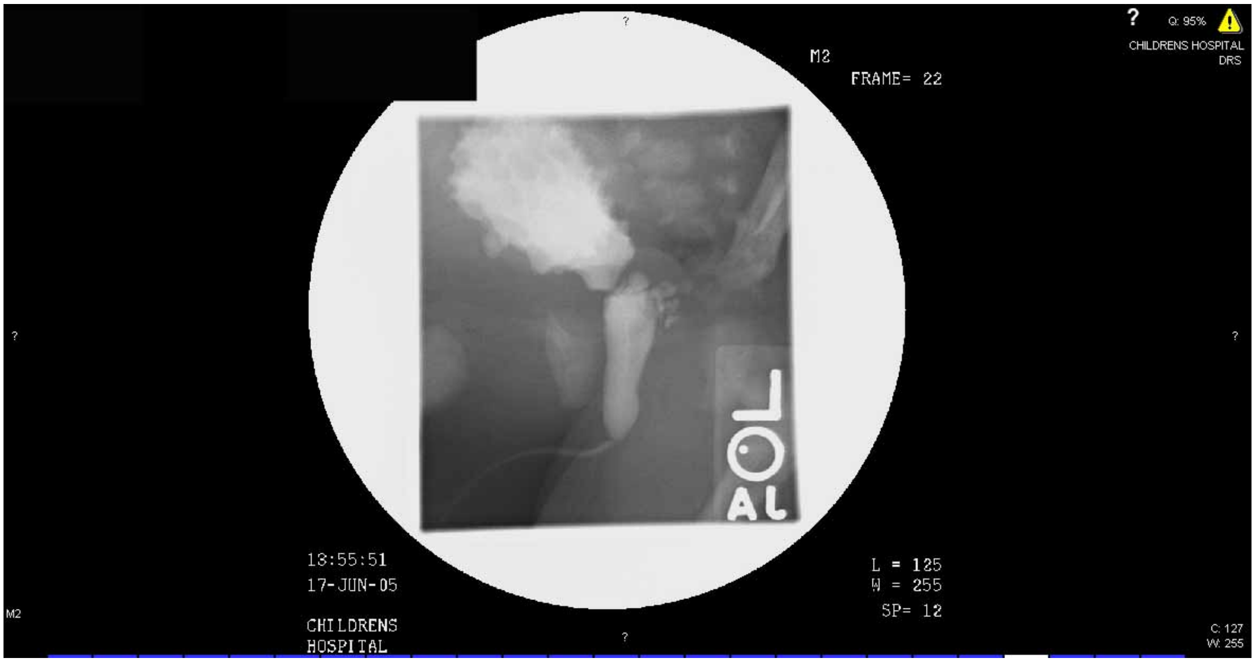

**FIGURE 6D.** VCU demonstrating a dilated prostatic urethra and a trabeculated bladder secondary to posterior urethral valves.

### Normal Postnatal Ultrasound

In the presence of a normal postnatal ultrasound, a repeat study is recommended at 4 weeks of age. If the second study is also normal, in the absence of VUR reflux, then no further imaging is necessary. The Brussels Free University Perinatal Nephrology Study Group reported a prospective review of patients with mild to moderate AH. In this review, 213 infants (139 boys, 74 girls) with antenatal mild to moderate hydronephrosis were followed at day 5, and months 1, 3, 6, 12, and 24. Eighty-one infants (38%) demonstrated a normal evaluation at birth. In all cases, the exam remained normal throughout the 24-month follow-up period[27]. However, delayed presentations of UPJ obstructions with normal postnatal ultrasounds have been reported and parents should be advised of this[18].

### Abnormal Postnatal Ultrasound

If the initial ultrasound is abnormal, then a repeat ultrasound is recommended at 1 month.

A contemporary standard does not exist for follow-up of SFU grades 1 and 2 hydronephrosis. Also, a controversy exists in the literature concerning the need for long-term follow-up of mild hydronephrosis. Interpretation of the literature is limited by the lack of agreement in terminology of hydronephrosis prior to implementation of the SFU grading system. In 2001, Sairam et al. evaluated the natural history of mid-trimester hydronephrosis. In their review of 268 women, mild (4–10 mm) hydronephrosis was seen in 80% of the fetuses. Of this group, none required surgical intervention postnatally. Furthermore, 88% of this cohort resolved the hydronephrosis in the antenatal or neonatal period[44]. In contrast, some reports raise questions as to the benign nature of mild pelvic dilation. Looking at the Brussels study of 213 infants with mild to moderate AH, significant uropathies were seen in 39% of the infants that required long-term follow-up, although intervention was required in only 3% of the patients[28]. In 1996, Morin et al. reported that surgical intervention for obstruction was required in 4 out of 63 patients who initially presented with minimal fetal hydronephrosis[37]. A recent report by Signorelli et al. demonstrated that up to 24% of patients with worsening of mild fetal pelvis dilation may require subsequent intervention[47]. In contrast, Odibo et al. reported an AP renal pelvis diameter threshold of 7 mm as predictive of normalization of postnatal studies[39].

Currently, most children with SFU grade 1 and 2 hydronephrosis are followed until they are mature enough to verbalize signs of distress. The ideal follow-up protocol for mild forms of hydronephrosis can only be resolved with multicenter prospective trial.

For SFU grade 3 or 4 hydronephrosis, a radionuclide renal scan may be needed. In the setting of bilateral hydronephrosis, the threshold for obtaining a radionuclide scan is much lower. Subsequent imaging studies for upper tract dilation should be based on the results of the VCU and the radionuclide renal scan (Fig. 2).

### Voiding Cystourethrogram

A lack of agreement exists concerning the need for a postnatal VCU in the presence of AH. The overall incidence of reflux in a population with AH ranges from 8–38%[10,26,56]. Factors such as the presence of ureteral dilation prenatally, the degree of pelvic dilation, race, and the presence of hydronephrosis have been reported to impact the results of the VCU. In favor of a selective approach, Moorthy et al. reported a 1% infection rate in patients evaluated with ultrasound alone for AH[36]. A second study by Yerkes et al. reported a similar finding in patients with SFU grade 2 hydronephrosis that they followed without performing a postnatal VCU. Intervention was not required in any of the 16 patients[53]. Horowitz et al. reported a significant variance in African-American compared to non-African-American patients and recommended selective screening for the non-African-American cohort[26]. Finally, Ismaili et al. reported that patients with two normal postnatal ultrasounds did not benefit from a VCU[28].

The literature is far more convincing in favor of indiscriminate postnatal VCU for hydronephrosis that is detected prenatally. A number of centers, including one multicenter review, have reported the initial postnatal ultrasound to be normal in up to 25% of patients with VUR that were identified with AH[24,50,56]. Herndon et al.[24] reported a majority of VUR to be high-grade, bilateral in male infants that demonstrated a normal initial postnatal ultrasound. Dudley et al.[57] reported that all of the patients with antenatally detected VUR demonstrated postnatal hydronephrosis of <10 mm. In addition, Herndon et al.[24] reported that 88% of patients with antenatal VUR demonstrated mild hydronephrosis postnatally. Some populations with AH may be at lower risk for VUR, such as the African-American population or female infants, however, currently there are no outcomes data to support the exclusion of a VCU in this population. Although controversies exist in the literature, VCU is strongly recommended for the fetus with AH regardless of the initial postnatal ultrasound, race, or gender of the infant. Antibiotic prophylaxis should be continued until the VCU is completed.

The timing of the postnatal VCU should be individualized with each patient. In cases of suspected bladder outlet obstruction, it is imperative to exclude this diagnosis within the first days of life. In cases of possible VUR, a VCU is recommended within 1 month of life. Although it would be ideal to exclude VUR as soon as possible in order to take these children off of prophylaxis, it is not practical for some families to do so because of transportation issues.

### Radionuclide Renal Scan

When concerns for obstruction exist, a "well-tempered diuretic Diethylene triamine pentaacetic acid (DTPA) or Mercaptoacetyltriglycine (MAG-3) renogram" should be performed[5]. This is a dynamic modality that provides relative function and drainage to aid the urologist in assessing the obstructive nature of AH. Of the two available agents, MAG-3 is preferred because of its favorable renal secretion properties. As with all studies, limitations exist and the efficacy of this modality varies significantly in the literature. Several important steps deserve mention. First, all patients should have a catheter placed in order to eliminate bladder pressure and prevent reflux of radionuclide. Second, lasix administration should be given with an appropriate dose at the point of maximal tracer uptake. Third, intravenous hydration is required in all patients. Despite these guidelines, significant variances have been reported. Some centers have demonstrated a significant alteration in relative function based on the size of the region of interest (ROI). The Great Ormond Street group reported that the relative variability of the ROI in the larger hydronephrotic kidney may explain the common finding of supranormal renal function[20]. In another study, Takla et al. reported on 51 patients with AH. Normalization of renal function and washout was demonstrated in 55%. The initial drainage curve was predictive of those patients who eventually normalized their studies. Normalization of renal drainage and function occurred in 86% of patients with an initial nonobstructive curve, 62% with an indeterminate scan, and 18% with an initial obstructive curve[49]. In contrast, Hafez et al. reported that the initial drainage curve from the initial renogram were not predictive of those patients requiring surgical intervention for UPJ obstruction[21].

The current recommendation for surgical intervention is an obstructive washout curve in which the T ½ exceeds 20 min or a significant discrepancy in split renal function (<40%) is detected. One caveat to split renal function is the patient with severe bilateral hydronephrosis. In this setting, split renal function is not an accurate indicator of overall renal function because of the absence of a normal control to compare to the hydronephrotic kidney. In this scenario, the renal unit that demonstrates the least function and, more importantly, the worst drainage should undergo repair. Although the indications for surgical intervention may appear straightforward, the drainage curve alone may be significantly altered if the child is dehydrated or lasix is given too early in the massively hydronephrotic kidney. In addition, the actual renal function may be significantly overrepresented in the large hydronephrotic kidney. Based on these inherent weaknesses, this author uses the renal scan to document baseline renal function and as a complement to the renal ultrasound more than an independent predictor of obstruction.

### MAGNETIC RESONANCE IMAGING UROGRAPHY (MRU)

As an imaging modality, MRU offers the advantages of providing superior imaging detail and functional assessment of obstruction without neonatal radiation exposure. The Emory group presented a recent report comparing MRU to conventional studies such as renal ultrasound and radionuclide renal scans. They concluded that MRU offered comparable results in terms of renal function and superior anatomic detail[40]. However, issues such as cost, availability, and the need for sedation in most patients significantly limits the widespread application of this imaging modality.

### POSTNATAL TREATMENT

It is beyond the scope of this text to evaluate critically the literature available for the entire differential diagnosis of AH. The most common disorders — including UPJ obstruction, multicystic dysplastic kidney (MCDK), VUR, ureterocele/ectopic ureter, primary megaureter, and posterior urethral valves — will be discussed.

Antibiotic prophylaxis should be administered until all studies have been completed. Two milliliters of amoxicillin daily (125 mg/5 cc) is preferred. Devastating infections have been reported when patients known to have AH are not appropriately managed postnatally[15]. Some studies have indicated that circumcision status may impact the infection rate in the presence of a known renal anomaly[7,24]. After 3 months, the Amoxil may be switched over to a nitrofurantoin (1/2 25-mg capsule) or trimethoprim sulfamethoxazole (1/2 tsp/10 kg).

#### Ureteropelvic Junction Obstruction

The initial enthusiasm for early intervention for UPJ obstruction that presented with AH recommended in the early 1980s has evolved over the last decade to a more conservative approach[30,31,41,51]. The most compelling data to support an initial interval of observation are the long-term follow-up by Koff et al. In this study, 104 newborns with unilateral severe hydronephrosis were managed expectantly. A deterioration of renal function was the indication for surgical repair. A total of 23 patients required intervention and a return of function was appreciated in all of these patients. In addition, hydronephrosis resolved in 69% and improved in 31%. The average time to this resolution was 2.5 years[51].

The initial report from the SFU reported on 33 surgeons representing 21 centers that included 582 cases of AH. Intervention was required in 41% of patients. In the intervention cohort, most patients demonstrated an obstructed renal scan and SFU grade 3 or 4 hydronephrosis. For the nonintervention cohort, grade 1 and 2 hydronephrosis predominated[34]. Although a consensus appeared to exist on characterizing those patients that underwent intervention, controversy still remains in identifying which of the patients with SFU grade 3 and 4 hydronephrosis go on to need intervention.

The most comprehensive series to date is the Great Ormond Street experience of AH. In a two-part series, they initially followed a standard protocol from 1980 to 1988. Patients with AH were divided into three groups based on differential renal function alone, <20%, 21–39%, and ≥40%. In the first group (<20% differential renal function), 3 of 10 kidneys that initially underwent pigtail drainage were successfully managed with surgical correction. In the second group (21–39% differential renal function), 23 of 28 underwent immediate pyeloplasty with 12 demonstrating an improvement in function and a stabilization of function in the remaining patients. The 5 patients who underwent observation, all improved on long-term follow-up without surgical intervention. For the last group (≥40%), none of the 34 kidneys with <12-mm pelvic dilation on the postnatal ultrasound required surgical intervention, compared to 27 of the 76 kidneys with pelvic dilation >12 mm that went on to have surgical correction of the obstruction[9].

Based on their observations, they set up a randomized trial, conservative vs. operative. The inclusion criteria was an AP renal pelvic diameter >15 mm postnatally, calyceal dilation, and differential function of ≥40%. Seventy-five patients (53 boys) were enrolled. Both groups were comparable in terms of mean hydronephrosis and differential function. At last follow-up, 39 patients underwent successful surgical intervention demonstrated by a decrease in hydronephrosis and an improvement in drainage. The mean function post-op of 50% compared favorably to the pre-op mean of 48%. One patient demonstrated a decrease in function from 43 to 38%. For the conservative group, 36 patients were randomized. Hydronephrosis resolved in 17 and improved significantly in 12. Surgery was required in 7 infants for renal deterioration that was recoverable in all but one patient. Again, AP diameter appeared to predict those patients requiring intervention. All 7 of the patients that crossed over to the intervention group demonstrated an AP diameter of >20 mm (20–40). However, 9 out of 17 of the patients with spontaneous resolution also initially demonstrated an AP diameter of >20 mm[9].

Relying on radiologic imaging alone may not allow adequate prediction of those renal units amenable to reconstruction. Elder compared renal biopsy data to pre-op renal scan differential function data. Interestingly, in the group with <40% differential function, 25% demonstrated essentially normal findings on renal biopsy. In the group with normal renal function, 21% demonstrated abnormal renal histology[12]. Based on these data, caution must be advised when interpreting differential function based on the “well-tempered renal scan”.

#### *Current Recommendations*

A practical approach should be followed in caring for the neonate with AH. All patients who are considered candidates for intervention should be placed on Amoxil prophylaxis, 2 cc (125 mg/cc) daily. In cases of grade 4 hydronephrosis, a repeat ultrasound and “well-tempered” renogram should be performed at 1 month. In the select case of profound delay in drainage or a demonstrative decrease in differential function, immediate intervention should be employed. In most patients, intervention can be deferred and a repeat ultrasound obtained at 3 months of age. If the renal ultrasound shows worsening or shows increased signs of distention, then a repeat “well-tempered” renal scan should be performed. Surgical intervention is reserved for an obstructive drainage curve or indeterminate curve with a deterioration in renal function (<40%). Close follow-up is mandatory for grades 3 and 4 hydronephrosis that are managed nonoperatively.

### Multicystic Dysplastic Kidney

The complete pathogenesis of MCDK is not clearly understood. However, most clinicians agree that MCDK represents the extreme spectrum of UPJ obstruction. The antenatal ultrasound of these two may be indistinguishable with the final diagnosis only being made postnatally. However, the findings of varying sizes of randomly located renal cysts, a large noncommunicating central cyst, and nonrenoform shape certainly are suggestive of MCDK. Associated renal anomalies can be seen in up to 43% of patients with contralateral UPJ obstruction and VUR being most common[1,2,22,52]. Indications for surgical removal include parental preference, hypertension, suspicious renal lesion, or urinary tract infections.

Surgical removal of the MCDK has been recommended because of concerns for increased incidence of Wilms’ tumor[25]. Most recent reviews indicate that previous reports were difficult to substantiate the diagnosis of MCDK. A large meta-analysis by Narchi involving 26 published reports failed to identify a single case of Wilms’ tumor in 1041 patients with MCDK[38]. Other indications for surgery include respiratory embarrassment and hypertension.

A cost-effective approach has been recommended by Perez et al. based on evidence-based outcomes studies. The two available options for observation include serial abdominal examinations or serial renal ultrasounds. Because Wilms' tumor manifests a rapid doubling time, it is recommended to perform ultrasound every 3 months until the age of 8. They concluded that nephrectomy was more cost effective than an observation protocol that involves serial ultrasounds. However, when observation involves only parental serial examination, then surgical removal no longer is cost effective[42].

#### Current Recommendations

Postnatally, a renal ultrasound confirms the presence of noncommunicating cysts. A VCU and (Dimercaptosuccinic acid) DMSA renal scan should be performed as well to rule out VUR and confirm the absence of function in the affected kidney (Figs. 7A,B). Clear indications for removal include suspicion of a tumor, hypertension, or respiratory compromise. Based on available data in the literature for an asymptomatic neonate with a MCDK, a reasonable approach is to offer the families the option of early nephrectomy or teach them self-examination. Although the risk of Wilms' tumor development is fourfold higher in the MCDK population, it does not warrant routine nephrectomy. When critically analyzed, serial ultrasounds are not cost effective either, but may be preferred by some practitioners.

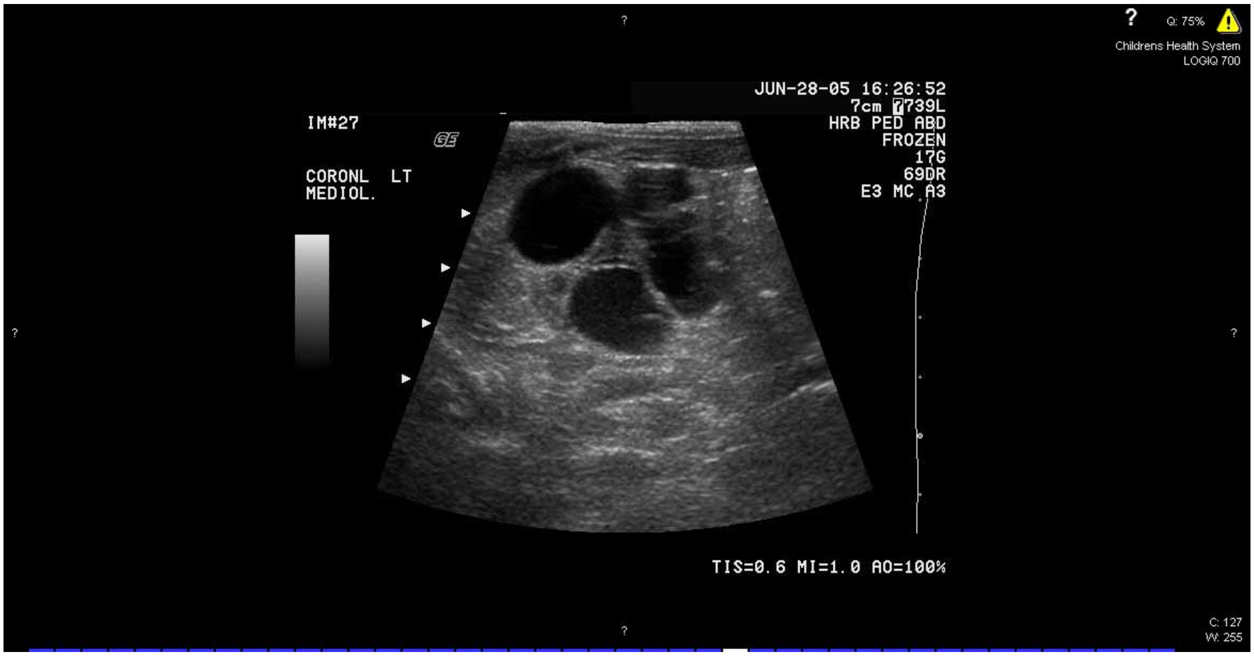

**FIGURE 7A**. Renal ultrasound demonstrating multiple noncommunicating cysts without parenchyma indicative of MCDK.

### Vesicoureteral Reflux

The natural history of reflux detected antenatally is quite different from reflux that presents with urinary tract infections. Antenatal reflux occurs predominately in boys and is high-grade and bilateral in most (Fig. 8). In addition, a significant number of these patients demonstrate renal dysplasia that appears to be developmental in origin[54]. Fortunately, antenatal reflux appears to have a steeper resolution curve in comparison to reflux that is detected postnatally. An explanation may, in part, be due to delayed maturation of the sphincter/bladder neck mechanism evidenced by elevated voiding pressures[13,24,54,55].

In 1999, Herndon et al. presented a multicenter report of VUR detected with AH[24]. A total of 71 (56 boys) patients representing 116 refluxing units were reviewed. High-grade VUR demonstrated a reflux resolution rate of 20% at a mean of 0.9 years in boys and 2.1 years in girls. Circumcision status significantly impacted the infection rate and need for surgical intervention despite antibiotic prophylaxis. They recommended an initial interval of medical observation in all patients regardless of the grade of VUR.

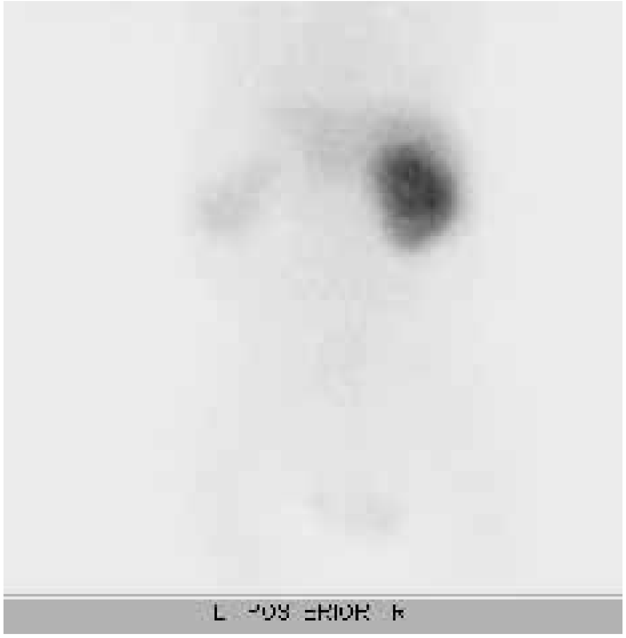

**FIGURE 7B**. Renal scan demonstrating the absence of function in a MCDK.

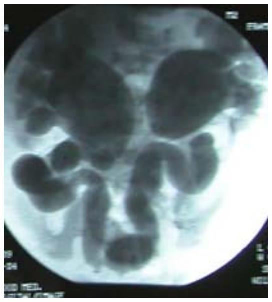

**FIGURE 8**. VCU demonstrating bilateral grade 5 VUR reflux.

### Current Recommendations

The physician caring for the child with VUR detected with AH should take a conservative approach. The child should be placed on Amoxil prophylaxis and transitioned over to nitrofurantoin or trimethoprim sulfamethoxazole prophylaxis at 3 months of age. A renal ultrasound should be performed every 6 months and VCU performed yearly. However, if indications for surgical intervention are met (i.e., breakthrough urinary tract infections), then corrective surgery should be performed. The surgical options include open ureteral reimplantation or cystoscopic subureteric injection.

### Duplication Anomalies (Ureterocele/Ectopic Ureter)

Renal duplication anomalies may present antenatally with hydronephrosis. A dilated ureter corresponding to the upper-pole moiety of the affected kidney is identified. The dilated ureter can be traced towards the urinary bladder where the identification of an ureterocele can be made by visualizing a cystic structure within the urinary bladder on ultrasound. The absence of this structure allows the presumptive diagnosis of an ectopic ureter to be made.

#### *Current Recommendations*

Postnatal imaging should include a VCU and radionuclide scan that can be done electively. Reflux is common in duplication anomalies especially in the laterally displaced, lower-pole moiety ureteral orifice. The renal scan aids in the assessment of function to the upper-pole moiety, which corresponds to the dilated ureter (Figs. 9A–C).

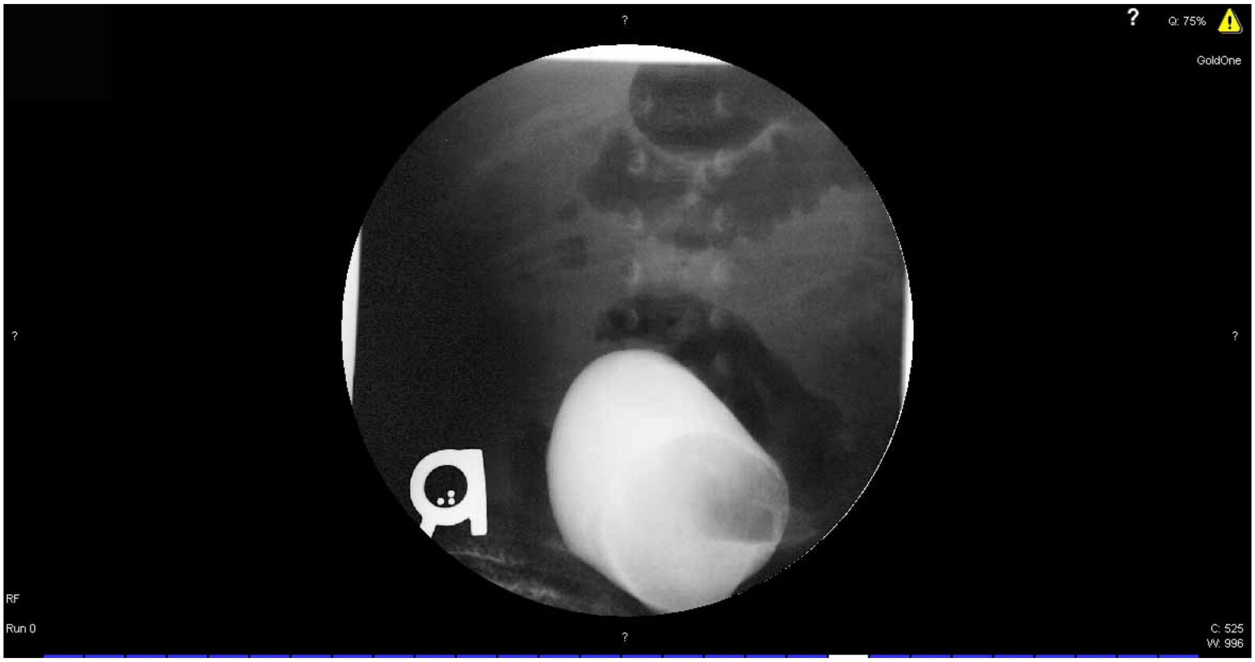

**FIGURE 9A**. VCU demonstrating shadow of ureterocele within the urinary bladder.

The plan for surgical intervention is based on the results of the VCU and renal scan. The surgical options include: cystoscopic puncture of the ureterocele, upper-pole heminephrectomy with excision of the dilated ureter, upper- to lower-pole ureteroureterostomy or ureteropyelostomy, or excision of the ureterocele and a common sheath reimplantation. There is no standard approach that can be applied to all patients and each intervention should be individualized. In general, if the baby is infected or the bladder is obstructed from the ureterocele, then incision of the ureterocele is performed.

If the upper pole shows no function on the renal scan, then an upper-pole heminephrectomy may be performed. Recently, however, some authors have presented data supporting the role of incision of the ureterocele with reimplantation of the nonfunctioning upper pole. The short-term follow-up of these patients is encouraging, however, long-term (>20 years) follow-up of the reimplanted nonfunctioning upper pole is lacking[19].

If the upper pole demonstrates function, then reimplantation of both ureters at the bladder level, upper- to lower-pole ureteroureterostomy at the kidney or bladder level, or incision of the ureterocele may be performed.

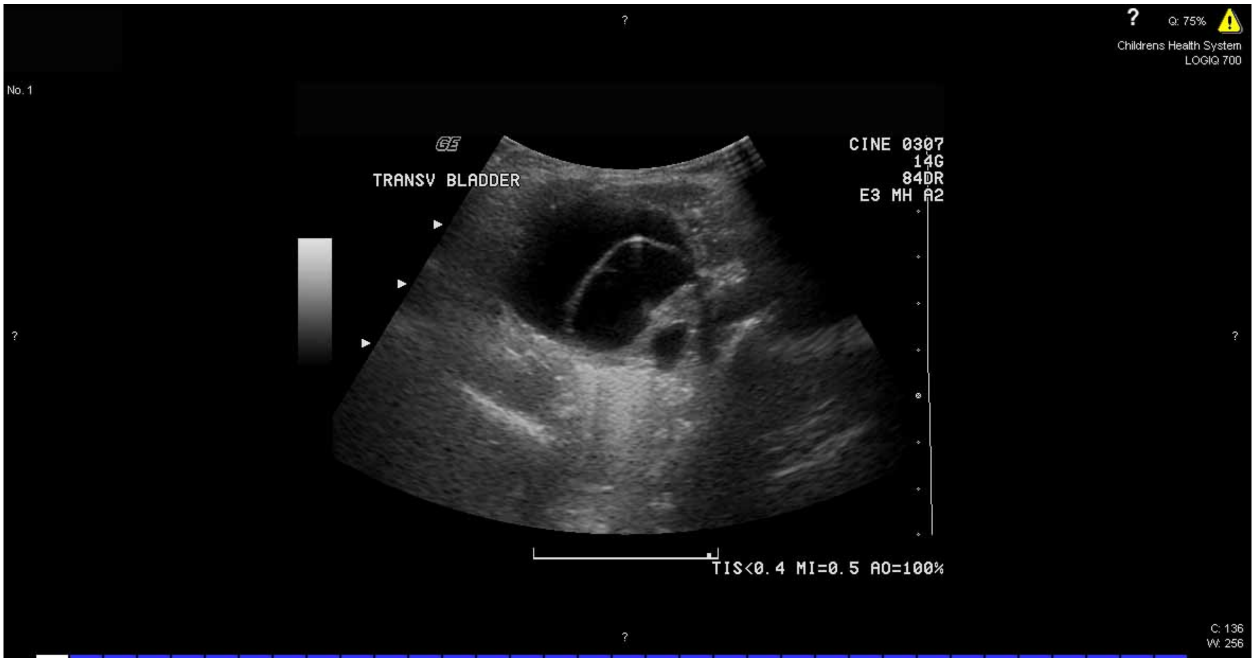

**FIGURE 9B**. Bladder ultrasound demonstrating ureterocele within the urinary bladder.

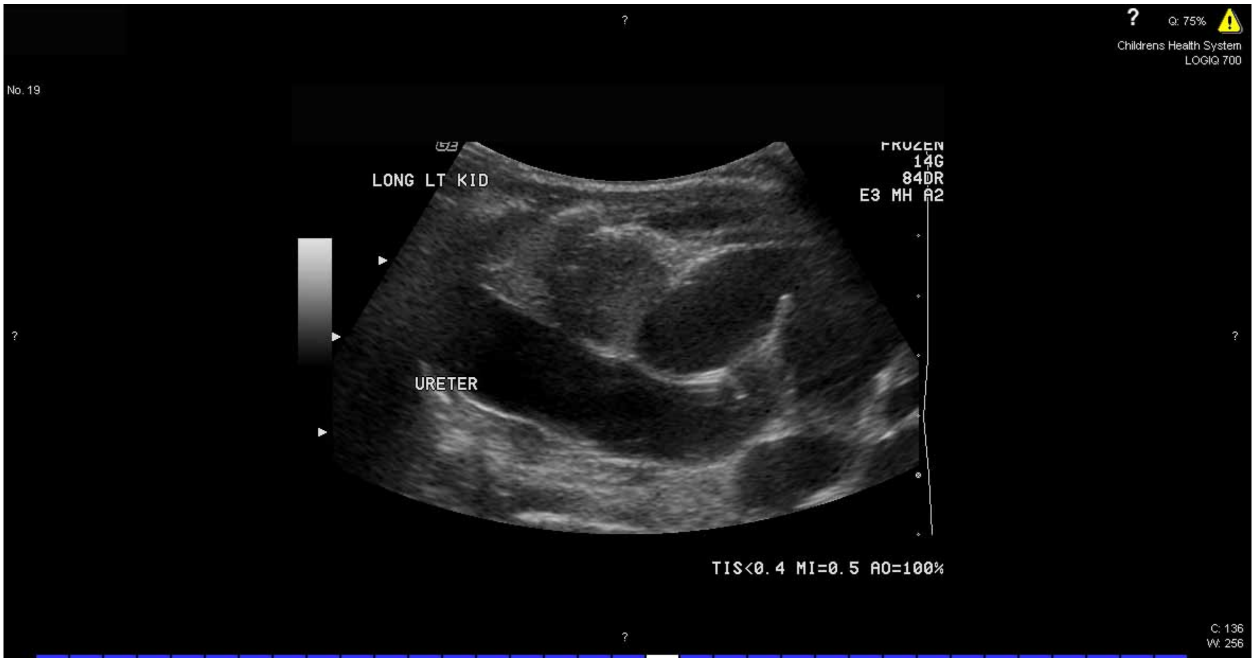

**FIGURE 9C**. Renal ultrasound demonstrating dilated upper-pole moiety ureter and the absence of parenchyma in the upper pole of the kidney.

### Megaureter

As with most entities that initially were detected asymptomatically in the antenatal period in the 1980s, patients with obstructed megaureters were initially managed aggressively from a surgical perspective. However, several recent reviews have demonstrated a consensus towards a more conservative approach to patients with antenatally detected megaureters[33,35,45]**.** In the Boston series of 69 nonrefluxing megaureters, initial grade of hydronephrosis was a significant predictor of resolution. Grade 1–3 hydronephrosis resolved at a mean of 13, 24, and 35 months, respectively, compared to an average of 49 months for less than grade 3 hydronephrosis. The Philadelphia series published the longest follow-up with a mean of over 6 years in 27 patients representing 40 megaureters. Complete resolution of hydronephrosis occurred in 52% of patients at an average of 2.9 years. Long-term follow-up was available for 10 patients at a mean of 13.4 years. Of this group, one patient deteriorated asymptomatically with a reduction of renal function and the development of a contralateral renal stone. None of the 4 patients with resolved hydronephrosis redeveloped dilation[45].

#### *Current Recommendations*

In order to classify the antenatally detected megaureter, a VCU and MAG-3 renal scan should be performed. Once the diagnosis of a nonrefluxing megaureter is made, then conservative medical observation should be first-line therapy. Antibiotic prophylaxis is generally recommended for severe hydronephrosis and should follow the guidelines outlined above. Indications for surgical correction include deterioration in renal function (<40%), infection, or pain. Based on the available literature, this should represent about 10–20% of the nonrefluxing megaureters.

### Posterior Urethral Valves

As previously mentioned, any male with bilateral hydronephrosis and a dilated or thickened urinary bladder should be worked up for the diagnosis of posterior urethral valves. A renal ultrasound and a VCU should be performed on the 1$^{st}$ day of life.

The initial management involves the placement of a feeding tube to decompress the obstructed bladder. The neonates should be closely monitored metabolically. When the creatinine nadirs, which may take several weeks, a repeat renal ultrasound should be obtained to ensure that the bladder and upper urinary tract are decompressed.

The decision to incise the valve is based, in part, on the overall renal function. In most patients, incision of the valve can be performed within the first few weeks of life. A number of approaches including cold hook knife, electrocautery, and balloon dilation are available. In select cases of profound upper tract dilation despite catheter drainage or severe renal failure, diversion of the bladder with vesicostomy or diversion of the ureters with ureterostomy/pyelostomy may be indicated.

### SUMMARY

In summary, following the inception of antenatal ultrasound, a two-stage paradigm shift has occurred in pediatric urology. Initially, the identification of a large number of asymptomatic infants appeared to afford the surgeon the opportunity for preemptive intervention. However, it has now become apparent that AH is far more difficult to interpret than originally perceived. The initial enthusiasm for surgery has now been replaced by a much more conservative approach to UPJ obstruction, MCDK, VUR reflux, and the nonrefluxing megaureter.

Postnatally, it is imperative to perform a comprehensive evaluation of the infant with a history of AH. The implementation of the Society for Fetal Urology grading system for hydronephrosis is encouraged at all institutions. Antibiotic prophylaxis should be administered shortly after birth. The initial renal ultrasound should be deferred until the 2nd day of life. Although the role for VCU is somewhat controversial, the available literature appears to support its indiscriminate application to all patients with AH. If bladder outlet obstruction is suspected, then the VCU should be performed within the first few days of life. In order to exclude VUR, the VCU should be performed within a practical time period within the 1st month of life. Radionuclide renal scans are used as a guide for intervention by documenting differential function and drainage of the severely hydronephrotic kidney.

Specific issues related to AH yet to be resolved that warrant future study include: appropriate follow-up of SFU grades 1–2 hydronephrosis in the absence of VUR, a consensus on which patients with SFU 3 and 4 hydronephrosis deserve surgical intervention, and a randomized trial for fetal intervention in the setting of bladder outlet obstruction.
